# Uptake of biosimilars in China: a retrospective analysis of the case of trastuzumab from 2018 to 2023

**DOI:** 10.1186/s41256-024-00372-z

**Published:** 2024-10-05

**Authors:** Qiyou Wu, Zhitao Wang, Yihan Fu, Ren Luo, Jing Sun

**Affiliations:** 1 School of Health Policy and Management, Chinese Academy of Medical Sciences & Peking Union Medical College, 5 Dongdansantiao, Dongcheng District, Beijing, China; 2 IQVIA China, 8F West Tower, Huijing Twin Towers, 12B Jianguomenwai Avenue, Chaoyang District, Beijing, China

**Keywords:** Biosimilars, Trastuzumab, Market dynamics, Market competitions, Uptake

## Abstract

**Background:**

The Chinese biosimilar industry has demonstrated rapid growth in recent years. Limited evidence is available about biosimilar uptake at the national level. This study aimed to assess biosimilar uptake in the case of trastuzumab and to explore potential factors influencing the biosimilar penetration at national and provincial levels.

**Methods:**

This study employed an interrupted time series analysis to assess the level and trend changes of national trastuzumab originator consumption and the overall trastuzumab consumption after the price reduction of the originator and the introduction of the first biosimilar using the China Hospital Pharmacy Audit procurement data from March 2018 to February 2023. A latent class trajectory model (LCTM) was also adopted to estimate the biosimilar penetration across 30 provincial-level administrative divisions (PLADs). Based on the LCTM grouping results, provincial characteristics were analyzed.

**Results:**

After rapid growth, the penetration of biosimilars demonstrated a moderate ascending trend at the national level, reaching 27% in February 2023. Following the introduction of the first biosimilar in July 2021, the consumption of the originator decreased by 0.5% per month (*P* = 0.008), and the growth rate of overall trastuzumab consumption decreased by 1.1% per month (*P* = 0.014). LCTM fit the best when the number of trajectory classes was two, dividing 30 PLADs into a group demonstrating a faster increase in biosimilar penetration and the other with a slower increase. The PLADs in the fast-increasing group had a higher proportion of the population covered by the national basic health insurance, a lower proportion of the urban population, a lower proportion of the population covered by the urban employee health insurance program, a lower gross domestic product per capita, a lower total health expenditure per capita, and a lower out-of-pocket expenditure.

**Conclusions:**

The uptake of trastuzumab biosimilars in China was lower compared with major European countries. The introduction of trastuzumab biosimilars presented a substitutional effect. Perceptions of physicians and patients, the medicines procurement model, competition from other biologics, and health insurance payment methods may influence biosimilar uptake. Enhancing a comprehensive understanding of biosimilars among physicians and patients, including biologics with biosimilars in the national pooled procurement, and implementing provider payment reforms could foster biosimilar penetration.

**Supplementary Information:**

The online version contains supplementary material available at 10.1186/s41256-024-00372-z.

## Background

Biological agents are essential in treating diseases that significantly impact patients’ health and quality of life, such as cancer and autoimmune diseases [1, 2]. However, the high costs of biologics impose a heavy economic burden on healthcare systems worldwide [3, 4]. In 2017, spending on biological agents in the United States accounted for 37% of total medicine costs of that year [5], which has risen to 46%, or about US$ 260 billion, in 2022 [6]. The global expenditure on biological agents reached US$452 billion in 2022 [7], with more than 30% of total spending on medicines in European Union (EU) countries towards biological agents [8, 9]. High prices for oncology medicines are raising concerns about the sustainability of healthcare systems across Europe [10, 11], and the situation is even worse in low- and middle-income countries, which struggle to fund biological medicines [1, 12–15]. It is necessary to establish a balance between biomedical innovation and the sustainability of healthcare systems. In this circumstance, biosimilars are emerging as a promising solution. A biosimilar refers to a biological medicinal product which is highly similar to a licensed biological agent (the reference product or originator) and has no clinical differences in quality, safety, and efficacy from the originator [2, 16]. Although biosimilars are generally cheaper than the originator, their price difference is not always as significant as the large gaps between generics and brand-name chemical products [17, 18].

Nevertheless, promoting biosimilar uptake in European countries has significantly reduced healthcare expenditures. Since the approval of the world’s first biosimilar in 2006, cumulative savings across EU countries have exceeded €18 billion [19–21]. Encouraging the clinical use of biosimilars can also foster market competition, forcing originators to reduce prices or offer more significant discounts. Cost savings from competition could enhance the accessibility of biological treatments and benefit more patients [19, 22, 23]. Improving the availability of biosimilars is especially crucial for low- and middle-income countries. In 2023, the World Health Organization (WHO) Model Lists of Essential Medicines, which regularly selects highly effective bio-products with prohibitively high prices, included two more biological agents targeting cancer and multiple sclerosis, partly due to the presence of their biosimilars that offer affordable treatment options for patients in low- and middle-income countries [24].

Despite the notable advantages of biosimilars in alleviating financial pressures on healthcare systems, existing research indicates that barriers still impede better uptake of biosimilars. Market penetration of some biosimilars exceeds 90% in Sweden but falls below 20% in Belgium [20, 25–28]. This disparity is linked to varying attitudes and perceptions among stakeholders such as physicians and patients toward biosimilars [29–31]. One significant barrier is the patients’ and physicians’ doubt about the safety and efficacy of biosimilars rising from a lack of information, which leads to negative attitudes and unwillingness [32–34]. For patients with a weaker ability-to-pay, their willingness to use biosimilars to save medical expenses is often more vital [35, 36]. Physicians are also more willing to recommend cheaper biosimilars to them, as suggested in studies conducted in China and Europe [29, 30]. The penetration of biosimilars is also highly influenced by the pricing, reimbursement, and procurement policies regarding biosimilar switching and substitution [20, 28, 37–39]. Reimbursement methods enabling hospitals and physicians to retain cost savings create financial incentives to utilize cheaper biosimilars [25–28, 37, 39].

The Chinese biosimilar industry commenced later than that of EU countries but has demonstrated a rapidly growing trend in recent years. Since 2019, when China’s first biosimilar was approved, a total of 49 biosimilars spanning 18 molecule entities have attained approval by the end of March 2024, of which 37 are monoclonal antibody medicines [40]. Additionally, there is a booming biosimilar research and development pipeline in China. However, unlike European countries, which have established and implemented comprehensive policies to promote the uptake of biosimilars, including financial incentives created by pricing, reimbursement, and provider payment methods, no direct policies have been explicitly formulated to promote biosimilar uptake in China. The national pooled procurement of insulin in 2022 played a crucial role in fostering market competition for insulin biosimilars [41]. This indicates an anticipation that more biosimilars may be included in the national medicines pooled procurement. Current analysis in China primarily focused on investigating the uptake patterns of biosimilars in individual hospitals or provincial-level administrative divisions (PLADs) [29, 42, 43]. Notably, there is a lack of research on the uptake of biosimilars at the national level.

Trastuzumab is the standard treatment for human epidermal growth factor receptor-2 (HER2) positive breast cancer and gastric cancer [44, 45]. Trastuzumab biosimilar is one of the first biosimilars approved in China and has already entered all provincial markets, indicating a broad observational period and a large sample size. Taking trastuzumab as an example, this study analyzed how the consumption of the trastuzumab originator changed before and after the introduction of the first biosimilar in China. We also analyzed the market penetration of trastuzumab biosimilars in 30 PLADs to identify potential influencing factors. The study aimed to provide evidence for promoting the clinical use of biosimilars and improving the accessibility of biological therapy, which would also help other resource-limited countries to address the issue of access to expensive bio-therapy.

## Methods

### Study design

The analysis was based on monthly time series consumption data of trastuzumab in Chinese hospitals at the national level. We conducted the interrupted time series (ITS) regression to quantify the level and trend changes in the consumptions of trastuzumab originator and overall trastuzumab (including both the originator and biosimilars) before and after the introduction of the first trastuzumab biosimilar. Based on the monthly panel data of biosimilar market penetration in 30 PLADs during the observation period, we adopted the latent class trajectory model (LCTM) to show provincial differences and identify potential factors that may affect biosimilar market penetration across PLADs. We also reviewed the timeline of the market authorization for bio-products with the same indications as trastuzumab and performed a descriptive analysis of the consumption volumes of these products during the observation time of September 2020 to March 2023, which might divert the market share of trastuzumab.

### Subjects and data source

This study took the trastuzumab originator and its biosimilars as a case. The trastuzumab originator was approved in 2002 and included in the national basic health insurance in 2017 following a substantial price reduction through national price negotiation [46]. Further price reductions were negotiated through a renewal of the price agreement in 2019, and the updated price was implemented nationwide in January 2020 [47]. The first trastuzumab biosimilar was approved in August 2020 and entered all provincial markets in July 2021. Currently, there are multiple biosimilars available in the market. Treatment cost for trastuzumab has been continuously diminished, from the private price of US$3500/440 mg to the first negotiated price of US$1100/440 mg and the current renewed price of US$800/440 mg for the originator. Current prices are US$240/150 mg and US$120/60 mg for biosimilars.

Cancer patients receiving trastuzumab treatment are typically hospitalized or treated in day wards, so trastuzumab is majorly dispensed by hospital pharmacies. This study adopted the IQVIA-China Hospital Pharmaceutical Audit (CHPA) dataset to measure the consumption of trastuzumab [48]. CHPA collects monthly medicine  consumption data from 2253 representative sample hospitals with over 100 beds in mainland China (including county-level hospitals in rural areas, which accounted for about 30% of the total number of hospitals with over 100 beds in the country) and offers projected medicine consumption data of 31 PLADs, which accounts for more than 70% of the national hospital medicine market. The observation period was from March 2018 to February 2023, which started before the renewed price of trastuzumab originator was implemented across all PLADs in January 2020 and after the first biosimilar was marketed in all PLADs in July 2021, comprising 60 months. PLADs with a missing data rate > 5% (Tibet) were excluded. Consumption data for trastuzumab emtansine and inetetamab was extracted from CHPA. We also retrieved provincial demographic and socio-economic data from the China Statistical Yearbook 2022 and the China Health Statistical Yearbook 2022 [49, 50].

### Measures

Given the consistent dosage across trastuzumab originator and biosimilars for each indication (including metastatic breast cancer, early-stage breast cancer, and metastatic gastric cancer) [51], we converted the consumption volumes of different products into milligrams for analysis. Due to its highly individualized feature, no defined daily dose (DDD) has been established for trastuzumab. We performed the same calculation of consumption volumes for similar bio-products with the same indications as trastuzumab. The outcome variables for the ITS analysis were the logarithm of trastuzumab originator consumption and the overall trastuzumab consumption volumes at the national level. The interventions for the ITS analysis were the price reduction resulting from the originator’s price agreement renewal in January 2020 and the uptake of the first biosimilar in all PLADs in July 2021. The outcome variable for the LCTM analysis was the market penetration of trastuzumab biosimilars, represented by the proportion of biosimilar consumption volume to the overall trastuzumab consumption volume. Considering that the penetration of biosimilars is highly associated with the ability-to-pay of patients, the healthcare systems, and policies regarding biosimilar switching and substitution [38, 39], the following socio-economic characteristics were compared across different groups of PLADs in the LCTM analysis, including the proportion of the urban population to total population, the proportion of the population covered by the national basic health insurance to the total population, the proportion of the population covered by urban employee health insurance program to total insured population, gross domestic product (GDP) per capita, total health expenditure (THE) per capita, and proportionate out-of-pocket (OOP%) expenditure. In addition, demographic characteristics of PLADs in terms of total population were also compared across different groups of PLADs.

### Statistical analysis

We developed an ITS linear regression model to analyze the level and trend changes in the consumption volumes of trastuzumab before and after two interventions: the price reduction of the originator in January 2020 and the uptake of the first biosimilar in July 2021. The time points of January 2020 and July 2021 were incorporated into the following ITS linear regression model:

$$\begin{aligned} Y_{t} & = \beta_{0} + \beta_{1} time_{t} + \beta_{2} intervention_{1t} + \beta_{3} time_{t} *intervention_{1t} \\ & \quad + \beta_{4} intervention_{2t} + \beta_{5} time_{t} *intervention_{2t} + \varepsilon_{t} \\ \end{aligned}$$

$time_{t}$ is a continuous variable indicating time in months at time *t* from the start of the observation period (March 2018); $time_{t}$ = 1, 2, 3, …, 60, there are 60 observation time points in total. $intervention_{1t}$ is an indicator for time *t* occurring before the intervention of price reduction of the originator (assigned as 0) or after intervention (assigned as 1) in January 2020. $intervention_{2t}$ is an indicator for time *t* occurring before the intervention of the introduction of the first biosimilar (assigned as 0) or after intervention (assigned as 1) in July 2021. The dependent variable $Y_{t}$ indicates the consumption of trastuzumab in month* t*. $\beta_{0}$ denotes the baseline (March 2018 to December 2019) level, i.e. average log mg per month, at time zero. $\beta_{1}$ denotes the baseline trend of outcome, i.e., change in the average log mg that occurred each month before the first intervention. $\beta_{2}$ and $\beta_{4}$ denote level changes in the average monthly log mg immediately after the interventions. $\beta_{3}$ and $\beta_{5}$ denote changes in the trend in the average monthly log mg after each intervention. The term $\varepsilon_{t}$ represents the random variability not explained by the model at time *t* [52].

We deployed the LCTM model to identify PLADs with similar market penetration rates of trastuzumab biosimilars and explore potential factors that might be associated with biosimilar uptake. LCTM is a special finite mixture model designed to identify latent classes of individuals following similar progressions of a determinant over time and simplify heterogeneous individuals into more uniform clusters [53, 54]. Our model used the second-order polynomial fitting. For each PLAD, we calculated the posterior probabilities for each trajectory and assigned PLADs to the trajectory with the highest probability. We estimated the best-fitting number of trajectories based on a minimum Bayesian Information Criterion (BIC) [55], while maintaining the posterior probabilities by trajectory class > 0.70 and class size ≥ 2% of the total number of PLADs [54]. The characteristics of PLADs in different trajectory classes were listed in the form of a thermal map.

For all analyses, a two-tailed *P* value < 0.05 was considered statistically significant. We performed all statistical analyses using the STATA 15.1 software (StataCorp LLC. 2017).

## Results

### Trastuzumab consumption and biosimilar uptake at the national level

**Fig. 1:**
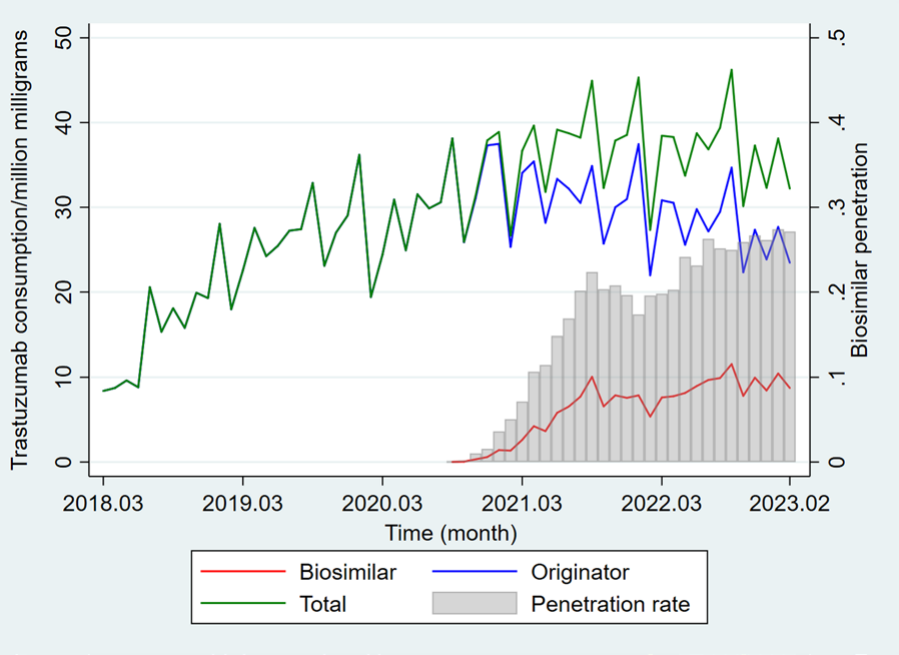
Trastuzumab monthly consumption and biosimilar penetration at the national level

At the national level, the overall trastuzumab consumption volume generally demonstrated a fluctuating upward trend during the observation. The growth rate slowed down in the later period. Following the introduction of the first trastuzumab biosimilar, the consumption of biosimilars continued to increase while that of the originator gradually declined. After an initial rapid growth, the national market penetration of biosimilars demonstrated a more moderate ascending trend, reaching 27% in February 2023 (Fig. 1).

### Level and trend changes in trastuzumab consumption volume at the national level after the introduction of the first biosimilar

**Fig. 2:**
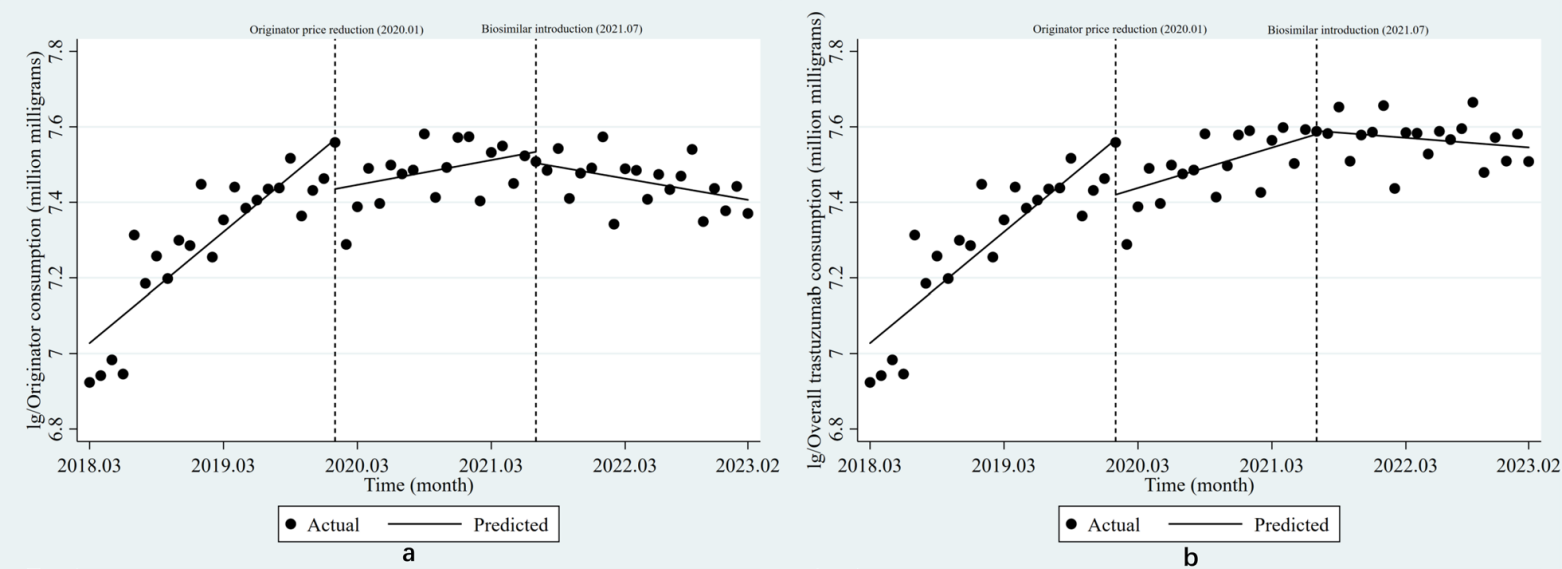
**a** ITS regression of trastuzumab originator monthly consumption volume at the national level. **b** ITS regression of overall trastuzumab monthly consumption volume at the national level

The results of the ITS analysis are shown in Table 1. From March 2018 to January 2020, the national consumption of the trastuzumab originator grew at a rate of 2.5% per month (*P* < 0.001). When the price of trastuzumab originator was reduced in January 2020, its consumption immediately decreased by 13.1% (*P* = 0.029), accompanied by a 1.9% decrease in growth rate (*P* = 0.001). Following the introduction of the first biosimilar in July 2021, the growth rate of the originator consumption further decreased by 1.1% (*P* = 0.019), and the consumption decreased by 0.5% per month (*P* = 0.008) (Fig. 2a).

**Table 1:** ITS regression results of trastuzumab monthly consumption at the national level

	Originator consumption			Overall trastuzumab consumption		
	*β* (95% *CI*)	SE	*P* value	*β* (95% *CI*)	SE	*P* value
Baseline level	7.028 (6.931 to 7.125)	0.048	**< 0.001**	7.028 (6.931 to 7.125)	0.048	**< 0.001**
Baseline trend	0.025 (0.017 to 0.032)	0.004	**< 0.001**	0.025 (0.017 to 0.032)	0.004	**< 0.001**
Level change after originator price reduction	− 0.131 (− 0.249 to − 0.014)	0.058	**0.029**	− 0.147 (− 0.264 to − 0.029)	0.059	**0.016**
Trend change after originator price reduction	− 0.019 (− 0.030 to − 0.008)	0.005	**0.001**	− 0.016 (− 0.026 to − 0.005)	0.005	**0.005**
Trend after originator price reduction	0.006 (− 0.003 to 0.014)	0.004	0.176	0.009 (0.001 to 0.017)	0.004	**0.031**
Level change after clinical introduction of the 1st biosimilar in all PLADs	− 0.030 (− 0.112 to 0.053)	0.041	0.472	0.009 (− 0.074 to 0.092)	0.041	0.829
Trend change after clinical introduction of the 1st biosimilar in all PLADs	− 0.011 (− 0.019 to − 0.002)	0.004	**0.019**	− 0.011 (− 0.020 to − 0.002)	0.004	**0.014**
Trend after clinical introduction of the 1st biosimilar in all PLADs	− 0.005 (− 0.009 to − 0.001)	0.002	**0.008**	− 0.002 (− 0.006 to 0.002)	0.002	0.227

*ITS* interrupted time series, *PLADs* provincial-level administrative divisions, Dickey–Fuller test *P* = 0.001 (originator consumption) and *P* = 0.006 (overall trastuzumab consumption); Durbin-Watson statistics = 2.17 (originator consumption) and 2.21 (overall trastuzumab consumption); values in bold are significant (*P* < 0.05)

From March 2018 to January 2020, the overall trastuzumab consumption volume showed an increasing trend, with an average monthly growth rate of 2.5% (*P* < 0.001). The overall consumption of trastuzumab immediately decreased by 14.7% after the price reduction of the originator (*P* = 0.016), and the growth rate declined by 1.6% (*P* = 0.005), maintaining a growth rate of 0.9% per month (*P* = 0.031). Following the introduction of the first biosimilars, the growth rate of overall consumption of trastuzumab decreased by 1.1% per month (*P* = 0.014) (Fig. 2b).

To check the robustness of the estimation, we performed sensitivity analysis by extending the time point of the second policy intervention to various milestones: when the consumption data of biosimilar was first available in September 2020 (in 6 PLADs), when the consumption data of biosimilar was available in 50% of the 30 PLADs (15 PLADs) in November 2020, and when the consumption data of biosimilar was available in 75% of the 30 PLADs (23 PLADs) in January 2021. The level changes and trend changes of the second policy intervention (clinical introduction of the first trastuzumab biosimilar) were all consistent, not statistically significant (Additional file 1: Tables S1–S3).

We also performed a sensitivity analysis to test for potential seasonal trends by including quarter dummies in the ITS regression model. No seasonal trend was found based on that analysis. The regression results were consistent with each other, and none of the coefficients for quarter dummies were statistically significant (Additional file 1: Table S4).

### Trajectory classes of market penetration of trastuzumab biosimilars at PLAD level

**Fig. 3:**
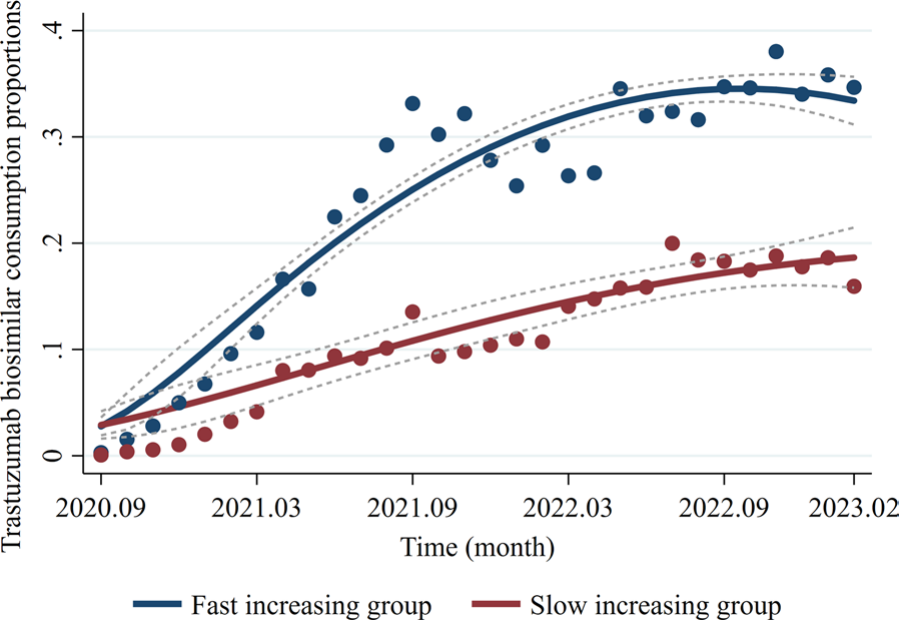
Class-specific mean predicted trajectory. *Notes*: The scatters are the actual mean consumption volume, the solid line is the fitting curve, and the dotted line area is the 95% confidence interval

The consumption volumes of trastuzumab originator and biosimilar at PLAD level are shown in Additional file 1: Fig. S1. The LCTM fit the best when the number of trajectory classes for biosimilar market penetration rate was two (Table 2), which divided 30 PLADs into one group demonstrating a fast increase in market penetration and the other group with a slow increase. The fast-increasing group included 16 PLADs (Anhui, Fujian, Guangdong, Guangxi, Guizhou, Hainan, Heilongjiang, Hunan, Jiangsu, Jiangxi, Qinghai, Shandong, Shaanxi, Shanghai, Sichuan, and Yunnan). The slow-increasing group included 14 PLADs (Beijing, Gansu, Hebei, Henan, Hubei, Jilin, Liaoning, Inner Mongolia, Ningxia, Shanxi, Tianjin, Xinjiang, Zhejiang, and Chongqing). The trajectory of the fast-increasing group demonstrated rapid growth in market penetration of biosimilars at the beginning of the observation period, slowed down after September 2021, and remained unchanged since September 2022. The trajectory of the slow-increasing group showed a tardy but continuous ascending market penetration of biosimilars (Fig. 3). During the observation period, the fast-increasing group consistently demonstrated a higher average market penetration than the slow-increasing group, though with slower growth after June 2022.

**Table 2:** Model parameter of LCTM analysis

Trajectories	BIC	Log-likelihood	AIC	Percentage of PLADs in groups
1				
2	770.28	783.88	775.88	53.3; 46.7
3	830.13	850.53	838.53	49.0; 27.3; 23.7
4	No convergence			

*LCTM* latent class trajectory models, *BIC* Bayesian information criterion, *AIC* akaike information criterion

**Fig. 4:**
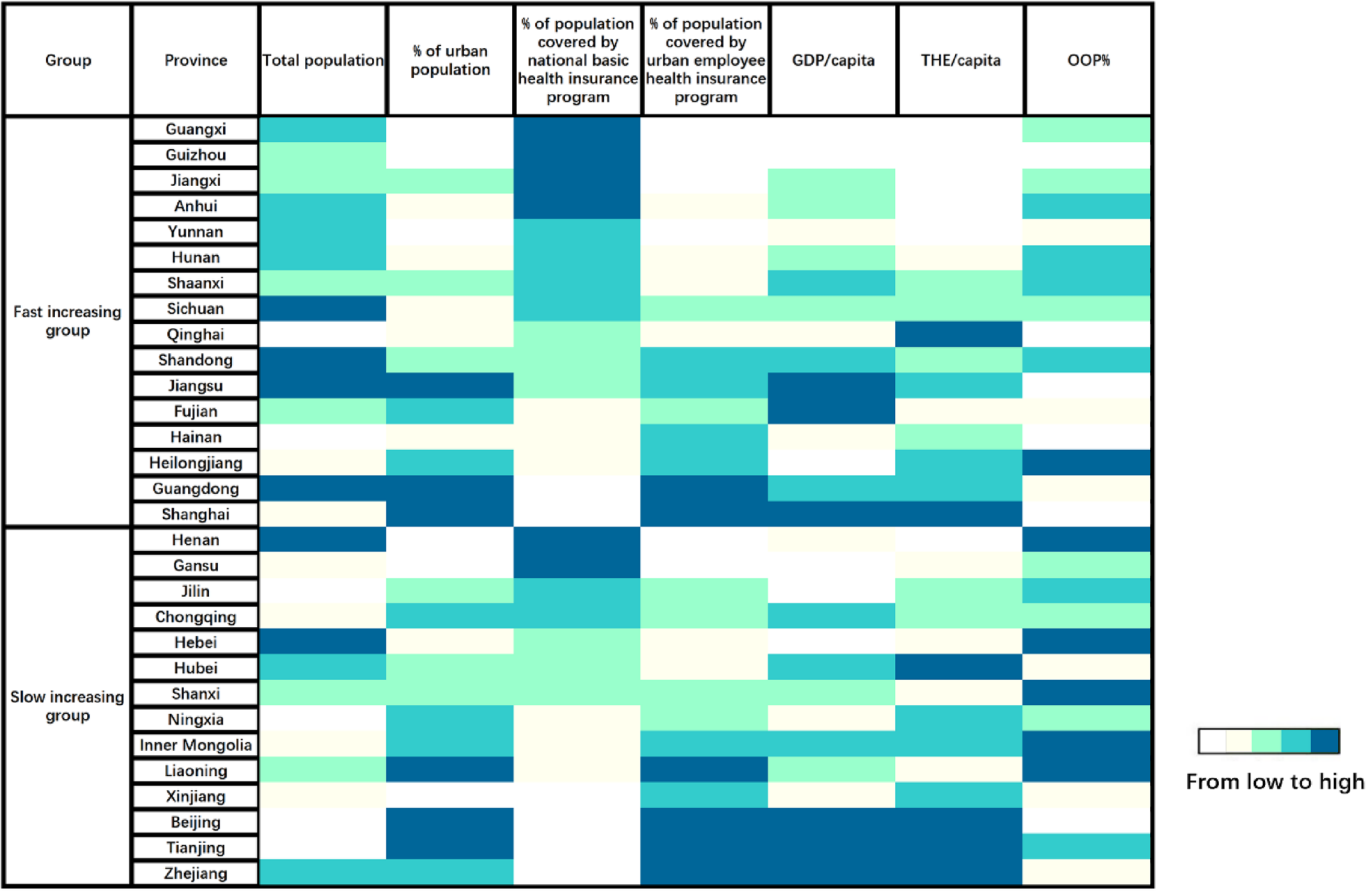
Thermal map of demographic and socio-economic characteristics of 30 PLADs in two trajectory classes. *Notes*: GDP = gross domestic product; THE = total health expenditure; OOP = proportionate out-of-pocket expenditure

The thermal map demonstrated differences in the demographic and socio-economic characteristics of the PLADs in two trajectory groups. More PLADs in the fast-increasing group had a higher proportion of the population covered by the national basic health insurance but a lower proportion of the urban population to the total population. They also had a lower proportion of the population covered by urban employee health insurance program to the total insured population, a lower GDP per capita, lower THE per capita, and lower OOP% (Fig. 4).

### Market authorization and consumption of similar products

Before February 2023, there were up to six entities of biological agents (pertuzumab, trastuzumab emtansine, inetetamab, disitamab vedotin, trastuzumab deruxtecan, and margetuximab) on the market with indications for HER2-positive breast cancer and gastric cancer, akin to trastuzumab. Among these drugs, pertuzumab, trastuzumab emtansine, inetetamab, and disitamab vedotin were included in the national basic health insurance (Additional file 1: Fig. S2). The national sales volume of inetetamab increased sharply after its introduction and reached US$1.4 million in February 2023. The national sales volume of trastuzumab emtansine primarily showed moderate growth, reaching the top of US$ 1 million in September 2021, and since then fluctuated around US$ 0.9 million (Additional file 1: Fig. S3).

## Discussion

### Market penetration of trastuzumab biosimilars

We found that following the introduction of the first trastuzumab biosimilar, the consumption volume of the originator decreased significantly and shifted from its upward trend to a gradual decline. In contrast, the overall consumption of trastuzumab remained stable. These findings suggest that the introduction of biosimilars led to a transition in treatment from the originator to the biosimilars. However, as of 2023, the average market penetration of biosimilar trastuzumab in China stood at only 27%. This level aligns with the evidence generated by other Chinese studies. A study conducted at a provincial cancer medical center reported that, among 446 patients diagnosed with HER2-positive breast cancer, 19.1% received treatment with trastuzumab biosimilars in 2021 [29]. Similarly, another study of 259 patients from Beijing diagnosed with non-small cell lung cancer, metastatic colorectal cancer, and hepatocellular carcinoma indicated that about 40% chose bevacizumab biosimilars in 2021 [43]. The market penetration of biosimilars in China is close to that of several developed countries in Asia and North America. In Japan, the market penetration of trastuzumab biosimilars was 13.4% in 2021 [56]. A study based on the National Health Insurance Service in South Korea reported a penetration rate of 13.9% for trastuzumab in 2018, and the average market shares of the second-generation biosimilars ranged between 20 and 25% [57]. In the United States, the market penetration of biosimilars exhibited significant variability, ranging from 5 to 60% in 2020, with an average of approximately 28% [58]. In 2021, except for bevacizumab and teriparatide, the market penetration of all other 14 biosimilars approved in Canada was about 20% [59, 60]. The biosimilar uptake in China remains lower compared to European countries, where most biosimilars hold market shares between 41 and 77%, showing a rapid increase over time [30, 37, 61, 62]. Noteworthy cases include Denmark, where the market share of biosimilars for trastuzumab reached 90% within three months of their introduction in 2018 and has since maintained around 95% [37].

Evidence shows that lower treatment expenditure is an essential factor affecting physicians’ and patients’ choice of biosimilars in most countries. When the price gap between biosimilars and the originator is narrow, it may dampen the willingness to adopt biosimilars [63, 64]. Following two rounds of national medicine price negotiations and price agreement renewals in China, the price of trastuzumab originator continued to decline, resulting in a small price gap between the originator and the biosimilars. Shifting to biosimilars could potentially lead to an average cost saving of US$421.11 per treatment course for HER2-positive breast cancer patients. Concurrently, the introduction of biosimilars also compelled the originator to reduce prices to maintain its current market share [64]. Multiple studies supported the notion that the introduction of biosimilars fosters market competition, pressuring the originator to lower prices or provide more significant discounts [8, 22, 23]. In some countries, the originator might adopt a very aggressive discount strategy to avoid losing market share, leading to a lower price than the biosimilars [62, 63].

The limited market penetration of trastuzumab biosimilars in China might be due to the relatively short time since the introduction. While the trastuzumab originator has been available in the Chinese market since 2002, the biosimilar has only been on the market for three years. Physicians have established a comparably mature understanding of the originator over years of clinical use. Consequently, they may not have fully embraced biosimilars regarding immunogenicity, safety, and interchangeability, which acts as a barrier to the uptake [30, 37, 39]. To address these challenges, healthcare authorities should enhance the disclosure of clinical and pharmacovigilance data on biosimilars, issue and enforce official clinical prescription guidelines for biosimilars, and, if viable, establish a specialized committee to equip physicians with sufficient information on biosimilars. These measures can enhance physicians’ understanding and recognition of biosimilars, and ultimately promoting their prescription of biosimilars [37, 39, 65].

### Procurement management model in public hospitals

Specific institutional arrangements within the healthcare system may also affect the market penetration of biosimilars. In Chinese public hospitals, only medicines listed in the hospital formulary can be procured through the regular process. If physicians decide to prescribe medicines off the hospital formulary, they have to submit special applications and acquire approval from the hospital’s drug therapeutic committee, which leads to inconvenience and weakens physicians’ willingness to use biosimilars [66]. However, in most public hospitals, adjusting the hospital formulary entails a lengthy administrative procedure, potentially impeding the timely clinical application of biosimilars [67]. Since trastuzumab biosimilars are relatively new to the market and not universally listed in hospital formularies, their uptake could be lagged by such institutional arrangements.

In contrast, most EU countries have implemented incentives to promote switch and substitution from originators to biosimilars. Nations like Germany, France, Norway, Sweden, and Denmark have introduced mandatory switching or stipulated the percentage of prescriptions dispensed with biosimilars. In these countries, the market penetration of biosimilars could exceed 70% [20, 37, 39, 63]. In Denmark, the national centralized procurement of hospital medicines forced physicians to choose successful biosimilar bidders. This approach saw the market penetration of infliximab and etanercept biosimilars reach 100% within a year of the introduction [39]. Procurement management practices in Chinese public hospitals should adapt to the increasing availability of biosimilars. Including more biological agents with approved biosimilars in the national medicines pooled procurement may help encourage hospital biosimilar procurement. More importantly, regulators within the healthcare authority and health insurance management agencies should establish incentive mechanisms to promote the clinical application of biosimilars. Wielding exclusive procurement for a swift biosimilar penetration has proven effective. Mandatory switch and quota on biosimilar prescription, although direct and quick in promoting biosimilar uptake, may not be easily implemented in a broader range since they are impractical in the current healthcare frameworks of most countries and regions out of Europe.

### Market competition from other biologics with the same indications

The ITS analysis revealed a deceleration in the overall consumption of trastuzumab after the price reduction of the originator, which further decreased after the introduction of the first biosimilar. This suggests that these two interventions did not promote the overall uptake of trastuzumab. Studies in European and Japanese markets have also observed a similar trend where the overall usage of various biologics decreased following the introduction of biosimilars. This phenomenon could be associated with the impact of the launch of a new generation of biologics with the same indications [8, 68]. Our study found that before February 2023, there were up to six entities of biological agents on the market with indications including HER2-positive breast cancer and gastric cancer, akin to trastuzumab. Notably, trastuzumab emtansine, approved in early 2020, can enhance precise target treatment and prolong the survival period in patients resistant to trastuzumab. On the other hand, inetetamab, introduced in June 2020, exhibits both targeting and immunotherapeutic effects and a more substantial effect of antibody-dependent cell-mediated cytotoxicity. Patients may choose these two innovative biological agents over trastuzumab. The continuous influx of competitive products could lead to a shrinking market share for trastuzumab. A study conducted in Nanjing city of Jiangsu found that trastuzumab emtansine, inetetamab, and pyrotinib had a substitutional effect on trastuzumab utilization [42].

### Socio-economic development factors

The LCTM analysis divided 30 PLADs into a fast-increasing group and a slow-increasing group based on their trastuzumab biosimilars’ penetration. The fast-increasing group has more PLADs with a lower GDP per capita, lower THE per capita, and lower OOP%, indicating weaker socio-economic development and stronger dependence on government subsidies. Former studies revealed a common tendency for patients with lower income and ability-to-pay to choose biosimilars for treatment over the originator due to their lower cost. One study in Portugal found that 61.7% of 108 patients with psoriasis were willing to use biosimilars for reduced individual expenses [35]. Similarly, a British study found that patients with Crohn’s disease considered lower medical costs was the primary reason for selecting biosimilars [36].

The fast-increasing group has more PLADs with a higher proportion of the population covered by the national basic health insurance and a lower proportion of the population covered by the urban employee health insurance program compared with the slow-increasing group, suggesting a weaker health insurance benefit. Chinese residents are generally entitled to two parallel national basic health insurance programs with separate funding collection and management mechanisms. The urban employee health insurance program offers more extensive benefits than the urban and rural resident health insurance program. The benefits provided are linked to the premium paid, and the premium of the resident program primarily relies on government subsidies for individuals with weaker ability-to-pay, which are substantially lower than those of the employee program. For patients with similar ability-to-pay, those enrolled in the resident program are more likely to choose biosimilars due to their weaker benefits [29]. Physicians are also more likely to recommend more affordable biosimilars to patients with inadequate health insurance coverage and limited economic capacities [30, 69, 70].

### Health insurance provider payment incentives

The divergence in market penetration of trastuzumab biosimilars across different PLADs may also be related to the evolving landscape of Diagnosis-Related Groups (DRG) payment reform in China. Unlike the traditional fee-for-service model based on the actual consumption of medical resources, DRG categorizes patients into specific disease groups based on disease severity, complexity of treatment, and homogeneity of medical resource consumption, and thus manages the treatment and payment of patients in the same disease group with the same method [71, 72]. Excess expenditure is no longer reimbursed, but the hospital can retain the balance [71, 73]. Through DRG payment reform, hospitals and physicians are incentivized to reduce costs by using cheaper biosimilars. PLADs in the fast-increasing group, including Guangxi, Guizhou, Heilongjiang, and Hunan, are all DRG demonstration sites designated by the health insurance, which may promote the market penetration of trastuzumab biosimilars.

Similar financial incentives to share savings generated from biosimilar uptake between health insurance providers and hospitals or individual physicians could be considered by regulators. In some regions of Italy, England, and Belgium, health insurance providers have developed financial incentives based on cost savings from biosimilar uptake [74–76]. Such shared savings can support hospital development or directly reward physicians, thus promoting biosimilar penetration in hospitals.

### Limitations

Firstly, the CHPA dataset only included consumption in hospitals, while consumption in community pharmacies was not included. The ‘dual-channel’ management mechanism implemented in China in 2021 allowed expensive medicines to be dispensed from the designated community pharmacies, which might divert some consumptions of trastuzumab from hospitals to the designated community pharmacies. However, trastuzumab is an injection for oncology treatment. Cancer patients under trastuzumab treatment are usually in the daytime ward of hospitals and would be more likely to have the medicines directly dispensed by the hospital pharmacies rather than the community pharmacies. This will help avoid potential risk factors throughout the distribution channel from the community pharmacies to the hospital ward. Thus, the potential underestimation of consumption is acceptable. Secondly, trastuzumab biosimilars entered the market for a short period in China, and the ongoing fluctuations in the trastuzumab market introduced constraints. Consequently, the observations are confined to a narrow timeframe. Thirdly, the analysis was restricted to a single biological agent alongside its biosimilars available in the market. Moreover, the study relied on provincial sales data rather than hospital-specific data that would provide insights into individual patient information. Given the complexity of health insurance policies like pricing and payment, the findings of this study should be carefully interpreted, and the conclusions should be carefully used to explain changes in the market share for other biological agents and their biosimilars. Fourthly, trastuzumab is indicated for HER2-positive breast cancer and gastric cancer, implying that the clinical transition to biosimilars may exhibit variations. Consumption data can not carry out investigations based on individual case studies to distinguish the above differences. Lastly, a more in-depth quantitative analysis is essential to gauge competitive biological agents’ influence on trastuzumab uptake. Future research on biosimilars in China should encompass a broader spectrum of biological agents and leverage data sets that furnish comprehensive insights into diseases, patients, and healthcare system characteristics, which will enable a more comprehensive analysis.

## Conclusions

Based on the consumption data of trastuzumab originator and biosimilars in 30 PLADs of mainland China, this study found that the uptake of trastuzumab biosimilars in China was lower than that observed in European countries. The introduction of the first trastuzumab biosimilar presented a substitutional effect. Factors affecting biosimilar uptake in China included formulary and procurement management models in public hospitals, market competition from biologics with the same indications, and health insurance payment methods. Policies and incentives in European countries to promote the uptake of biosimilars are yet well shaped in China. Healthcare authorities should enhance the disclosure of clinical data on biosimilars and provide official clinical prescription guidelines. Enhancing a comprehensive understanding of biosimilars among physicians and patients, including biological agents with biosimilars in the national pooled procurement, and implementing provider payment reforms could stimulate competition and foster the market penetration of biosimilars. These measures are also meaningful for other resource-limited countries to promote biosimilar uptake and address the issue of access to expensive bio-therapy.

## Supplementary Information

Additional file 1.

## Data Availability

The data that support the findings of this study are available from IQVIA Inc. Restrictions apply to the availability of these data, which were used under license for this study. Data are available with the permission of IQVIA Inc.

## Abbreviations

EU: European Union
WHO: World Health Organization
PLAD: Provincial-level administrative division
HER2: Human epidermal growth factor receptor-2
ITS: Interrupted time series
LCTM: Latent class trajectory model
CHPA: China Hospital Pharmaceutical Audit
DDD: Defined daily dose
GDP: Gross domestic product
THE: Total health expenditure
OOP: Out-of-pocket
BIC: Bayesian Information Criterion
CI: Confidence intervals
std. error: Standard error
DRG: Diagnosis-Related Groups
