# Supplementary material for: Uptake of biosimilars in China: a retrospective analysis of the case of trastuzumab from 2018 to 2023
Source: Glob Health Res Policy. 2024 Oct 5;9:42. doi: 10.1186/s41256-024-00372-z (PMC11453021; doi:10.1186/s41256-024-00372-z)

**Table S1** ITS regression results of trastuzumab consumption

with the biosimilar entering the first provincial market as its introduction

|  | **Originator consumption** | | | **Overall trastuzumab consumption** | | |
| --- | --- | --- | --- | --- | --- | --- |
|  | ***β* (95% *CI*)** | **Std. error** | ***P*-value** | ***β* (95% *CI*)** | **Std. error** | ***P*-value** |
| Baseline level | 7.028 (6.931~7.125) | 0.048 | **<0.001** | 7.028 (6.931~7.125) | 0.048 | **<0.001** |
| Baseline trend | 0.025 (0.017~0.032) | 0.004 | **<0.001** | 0.025 (0.017~0.032) | 0.004 | **<0.001** |
| Level change after originator price reduction | -0.147 (-0.324~0.031) | 0.088 | 0.103 | -0.147 (-0.324~0.031) | 0.088 | 0.103 |
| Trend change after originator price reduction | -0.017 (-0.048~0.015) | 0.016 | 0.293 | -0.017 (-0.048~0.015) | 0.016 | 0.293 |
| Trend after originator price reduction | 0.008 (-0.023~0.038) | 0.015 | 0.607 | 0.008 (-0.023~0.038) | 0.015 | 0.607 |
| Level change after clinical introduction of the 1^st^ biosimilar in the first province | 0.048 (-0.055~0.151) | 0.051 | 0.359 | 0.059 (-0.046~0.163) | 0.052 | 0.266 |
| Trend change after clinical introduction of the 1^st^ biosimilar in the first province | -0.012 (-0.043~0.019) | 0.015 | 0.442 | -0.007 (-0.038~0.024) | 0.015 | 0.655 |
| Trend after clinical introduction of the 1^st^ biosimilar in the first province | -0.004 (-0.007~-0.001) | 0.001 | **0.004** | 0.001 (-0.002~0.004) | 0.001 | 0.481 |

**Notes:** ITS=interrupted Time Series; the time point of biosimilar entering the first provincial market (September 2020) chosen as the biosimilar introduction; values in bold are significant (*P*<0.05).

**Table S2** ITS regression results of trastuzumab consumption

with the biosimilar entering 50% of all provincial markets as its introduction

|  | **Originator consumption** | | | **Overall trastuzumab consumption** | | |
| --- | --- | --- | --- | --- | --- | --- |
|  | ***β* (95% *CI*)** | **Std. error** | ***P*-value** | ***β* (95% *CI*)** | **Std. error** | ***P*-value** |
| Baseline level | 7.028 (6.931~7.125) | 0.048 | **<0.001** | 7.028 (6.931~7.125) | 0.048 | **<0.001** |
| Baseline trend | 0.025 (0.017~0.032) | 0.004 | **<0.001** | 0.025 (0.017~0.032) | 0.004 | **<0.001** |
| Level change after originator price reduction | -0.144 (-0.304~0.015) | 0.080 | 0.076 | -0.144 (-0.304~0.015) | 0.080 | 0.076 |
| Trend change after originator price reduction | -0.017 (-0.042~0.008) | 0.013 | 0.188 | -0.017 (-0.042~0.008) | 0.013 | 0.189 |
| Trend after originator price reduction | 0.008 (-0.016~0.032) | 0.012 | 0.520 | 0.008 (-0.016~0.032) | 0.012 | 0.517 |
| Level change after clinical introduction of the 1^st^ biosimilar in 50% of all provinces | 0.031 (-0.096~0.158) | 0.063 | 0.625 | 0.056 (-0.072~0.183) | 0.064 | 0.387 |
| Trend change after clinical introduction of the 1^st^ biosimilar in 50% of all provinces | -0.012 (-0.036~0.012) | 0.012 | 0.317 | -0.008 (-0.032~0.017) | 0.012 | 0.537 |
| Trend after clinical introduction of the 1^st^ biosimilar in 50% of all provinces | -0.004 (-0.007~-0.002) | 0.001 | **0.001** | 0.0003 (-0.003~0.003) | 0.001 | 0.818 |

**Notes:** ITS=interrupted Time Series; the time point of biosimilar entering 50% of all provincial markets (15 provinces, November 2020) chosen as the biosimilar introduction; values in bold are significant (*P*<0.05).

**Table S3** ITS regression results of trastuzumab consumption

with the biosimilar entering 75% of all provincial markets as its introduction

|  | **Originator consumption** | | | **Overall trastuzumab consumption** | | |
| --- | --- | --- | --- | --- | --- | --- |
|  | ***β* (95% *CI*)** | **Std. error** | ***P*-value** | ***β* (95% *CI*)** | **Std. error** | ***P*-value** |
| Baseline level | 7.028 (6.931~7.125) | 0.048 | **<0.001** | 7.028 (6.931~7.125) | 0.048 | **<0.001** |
| Baseline trend | 0.025 (0.017~0.032) | 0.004 | **<0.001** | 0.025 (0.017~0.032) | 0.004 | **<0.001** |
| Level change after originator price reduction | -0.152 (-0.296~-0.008) | 0.072 | **0.040** | -0.153 (-0.297~-0.009) | 0.072 | **0.038** |
| Trend change after originator price reduction | -0.015 (-0.033~0.004) | 0.009 | 0.114 | -0.014 (-0.032~0.004) | 0.009 | 0.127 |
| Trend after originator price reduction | 0.010 (-0.007~0.027) | 0.008 | 0.236 | 0.010 (-0.006~0.027) | 0.008 | 0.220 |
| Level change after clinical introduction of the 1^st^ biosimilar in 75% of all provinces | -0.013 (-0.117~0.091) | 0.052 | 0.806 | 0.025 (-0.082~0.132) | 0.053 | 0.647 |
| Trend change after clinical introduction of the 1^st^ biosimilar in 75% of all provinces | -0.014 (-0.031~0.003) | 0.008 | 0.095 | -0.010 (-0.028~0.007) | 0.009 | 0.226 |
| Trend after clinical introduction of the 1^st^ biosimilar in 75% of all provinces | -0.004 (-0.008~-0.001) | 0.002 | **0.005** | -0.0001 (-0.003~0.003) | 0.002 | 0.969 |

**Notes:** ITS=interrupted Time Series; the time point of biosimilar entering 75% of all provincial markets (23 provinces, January 2021) chosen as the biosimilar introduction; values in bold are significant (*P*<0.05).

**Table S4** ITS regression results of trastuzumab monthly consumption with quarter dummies

|  | **Originator consumption** | | | **Overall trastuzumab consumption** | | |
| --- | --- | --- | --- | --- | --- | --- |
|  | ***β* (95% *CI*)** | **Std. error** | ***P*-value** | ***β* (95% *CI*)** | **Std. error** | ***P*-value** |
| Baseline level | 7.023 (6.928~7.119) | 0.047 | **<0.001** | 7.021 (6.925~7.116) | 0.047 | **<0.001** |
| Baseline trend | 0.025 (0.019~0.031) | 0.003 | **<0.001** | 0.025 (0.019~0.031) | 0.003 | **<0.001** |
| Level change after originator price reduction | -0.136 (-0.247~-0.024) | 0.055 | **0.018** | -0.152 (-0.264~-0.040) | 0.056 | **0.009** |
| Trend change after originator price reduction | -0.018 (-0.028~-0.008) | 0.005 | **0.001** | -0.015 (-0.025~-0.005) | 0.005 | **0.006** |
| Trend after originator price reduction | 0.006 (-0.002~0.015) | 0.004 | 0.124 | 0.010 (0.001~0.018) | 0.004 | **0.022** |
| Level change after clinical introduction of the 1^st^ biosimilar in all provinces | -0.057 (-0.148~0.033) | 0.045 | 0.211 | -0.018 (-0.107~0.072) | 0.045 | 0.698 |
| Trend change after clinical introduction of the 1^st^ biosimilar in all provinces | -0.010 (-0.019~-0.001) | 0.004 | **0.025** | -0.011 (-0.020~-0.002) | 0.004 | **0.020** |
| Trend after clinical introduction of the 1^st^ biosimilar in all provinces | -0.004 (-0.008~-0.000) | 0.002 | **0.048** | -0.001 (-0.005~0.003) | 0.002 | 0.586 |
| Quarter dummies (Q1 as the reference) |  |  |  |  |  |  |
| Q2 | -0.016 (-0.080~0.049) | 0.032 | 0.629 | -0.011 (-0.074~0.053) | 0.032 | 0.739 |
| Q3 | 0.049 (-0.014~0.113) | 0.032 | 0.127 | 0.052 (-0.011~0.114) | 0.031 | 0.106 |
| Q4 | -0.020 (-0.082~0.042) | 0.031 | 0.523 | -0.021 (-0.083~0.041) | 0.031 | 0.498 |

**Notes:** ITS=interrupted Time Series; values in bold are significant (*P*<0.05).

**Figure
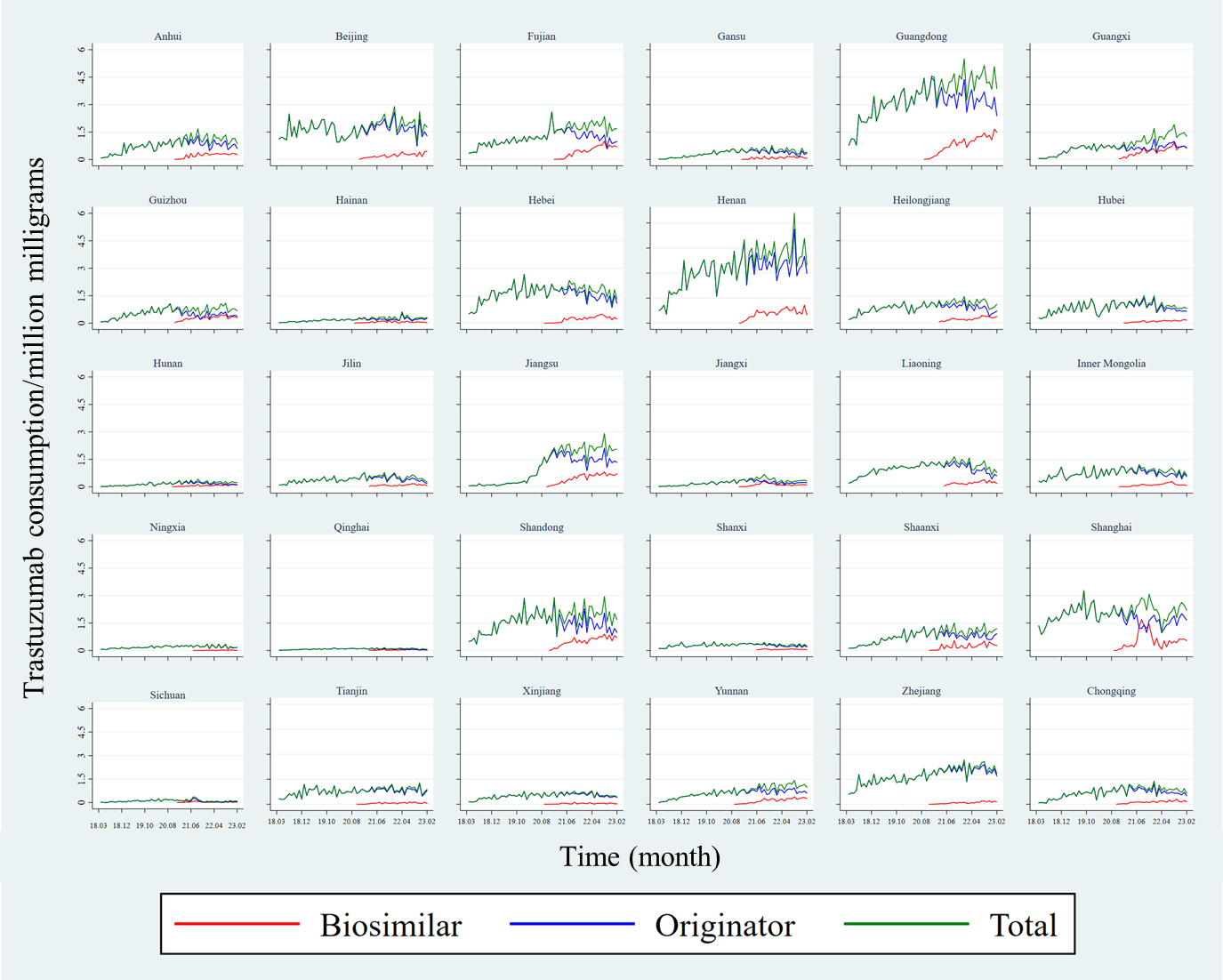
S1** Trastuzumab monthly consumption at the provincial level


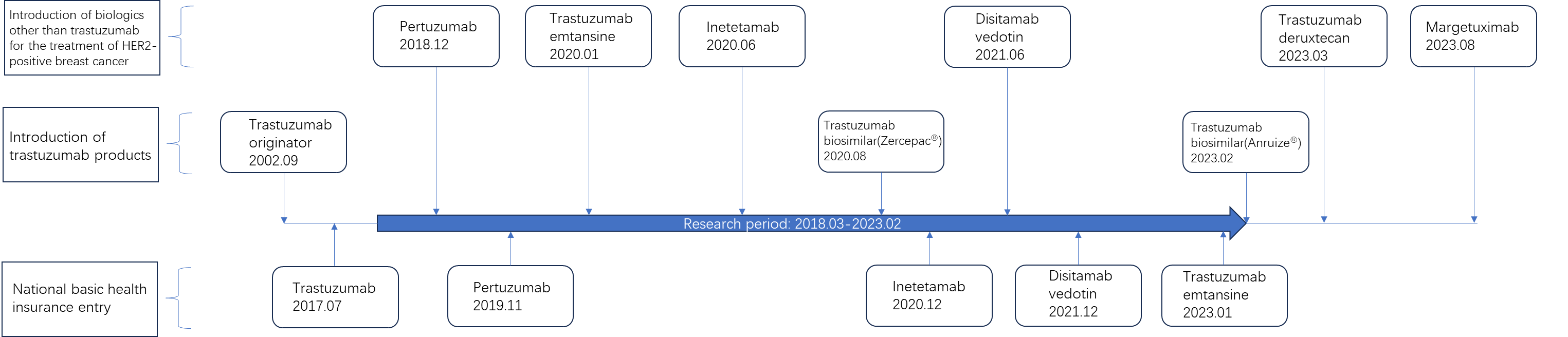


**Figure S2** Biologics for the treatment of HER2-positive breast cancer and gastric cancer in China

**Figure S3** Volume (million USD$) of Inetetamab and Trastuzumab emtansine in China
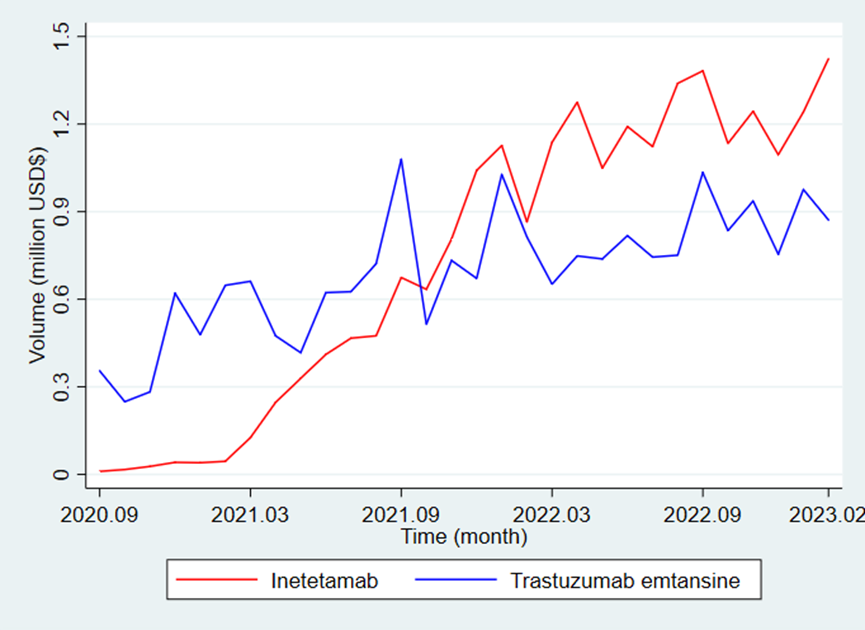

Supplement: Supplementary file 1 — Additional file 1. [file 41256_2024_372_MOESM1_ESM.docx]
